# Primary care metronidazole prescription in public and private facilities of South Benin: a register-based cross-sectional study

**DOI:** 10.1186/s12879-026-13717-5

**Published:** 2026-06-09

**Authors:** Harold Tankpinou Zoumenou, Dissou Affolabi, Rodrigue Kohoun, Léna Sandjakian, Jean-François Faucher

**Affiliations:** 1 Clinical Research Institute of Benin (IRCB), Abomey-Calavi, Benin; 2 Supranational Reference Laboratory for Tuberculosis, Cotonou, Benin; 3 Laboratory of Epidemiology of Chronic and Neurological Diseases, Cotonou, Benin; 4 Epimact: Inserm U1094, IRD UMR270, Univ. Limoges, CHU Limoges, EpiMaCT – Epidemiology of Chronic Diseases in Tropical Zone, Institute of Epidemiology and Global Health – Michel Dumas, OmegaHealth, Limoges, France; 5 Department of Infectious Diseases and Tropical Medicine, University Hospital of Limoges, 2 Avenue Martin Luther King, Limoges, 87000 France; 6 Coordination of Abomey-Calavi–So-Ava Health Zone, Ministry of Health, Abomey- Calavi, Benin; 7 Sorbonne Université, Inserm, Institut Pierre-Louis d’épidémiologie et de santé publique, Paris, F-75012 France

**Keywords:** Antimicrobials, Primary care, Public and private facilities, Low-income countries, Metronidazole

## Abstract

**Background:**

Metronidazole (MTZ) is a first-line antibiotic for several enteric infections. Its use is common in low-income countries, where most primary-care consultations are conducted by nurses. However, increasing resistance among some enteric pathogens is a growing concern. Using WHO guidelines, we conducted a retrospective study to assess MTZ prescribing practices and their determinants in public and private primary healthcare facilities in South Benin.

**Methods:**

We performed a register-based cross-sectional study covering the year 2020 in 11 primary healthcare facilities (5 public and 6 private) in Abomey-Calavi, South Benin, following WHO recommendations. In total, 200 visits per facility were selected using systematic random sampling. The primary outcome was the prevalence of MTZ prescription. Determinants of MTZ prescription were identified using multivariable logistic regression analysis.

**Results:**

In total, 2,200 medical visits were analyzed. The median age of patients was 19 years, and 57% were female. Antimalarials were prescribed in 52% of visits. Antibacterial agents were prescribed in the majority of visits, with MTZ being the second most frequently prescribed antibiotic (18%), after aminopenicillins (27%). In multivariable analysis, digestive symptoms (adjusted odds ratio [aOR], 8.86; 95% confidence interval [CI], 6.64–12.0), genitourinary symptoms (aOR, 6.65; 95% CI, 3.09–14.7), and skin lesions (aOR, 2.33; 95% CI, 1.54–3.51) were independently associated with increased odds of MTZ prescription. In contrast, fever (aOR, 0.64; 95% CI, 0.48–0.85), respiratory symptoms (aOR, 0.43; 95% CI, 0.26–0.70), and malaria (aOR, 0.21; 95% CI, 0.15–0.28) were associated with decreased odds. Male sex (aOR, 1.35; 95% CI, 1.05–1.73) and visits in the private sector (aOR, 2.31; 95% CI, 1.78–3.02) were also associated with higher odds of MTZ prescription.

**Conclusion:**

MTZ is the second most commonly prescribed antibiotic in primary care in the study area, with its use largely driven by digestive symptoms. Further studies are needed to assess the appropriateness of this prescription. Additionally, research is warranted to understand better the determinants of higher antimicrobial prescribing in the private healthcare sector.

**Supplementary Information:**

The online version contains supplementary material available at 10.1186/s12879-026-13717-5.

## Highlights

MTZ is the second most prescribed antibiotic in the study area.

MTZ prescription is primarily driven by digestive symptoms.

The private healthcare sector is independently associated with higher MTZ prescription rates.

Antimicrobial prescribing is generally higher in private healthcare facilities than in public facilities.

**Supplementary Information:**

The online version contains supplementary material available at 10.1186/s12879-026-13717-5.

## Background

Combating antimicrobial resistance (AMR) is a major challenge of the 21st century. It has been estimated that 1.14 million deaths were directly attributable to AMR in 2021, of which 209,000 occurred in Sub-Saharan Africa [1]. Beyond mortality and disability, AMR imposes substantial economic burdens, partly due to prolonged hospital stays [2]. In primary care settings, AMR is expected to result in an increasing number of infections that are either untreatable or costlier and more difficult to manage [3].

Metronidazole (MTZ) is an antibiotic classified within the World Health Organization (WHO) access group under the Access, Watch, Reserve (AWaRe) antibiotic categorization, which includes first- or second-line agents that provide optimal therapeutic value while minimizing the risk of resistance [4]. According to the WHO Model List of Essential Medicines, MTZ is indicated as a first-line treatment for intestinal infections caused by Clostridioides difficile, trichomoniasis, intra-abdominal infections (such as peritonitis), and necrotizing fasciitis [5]. As MTZ is a first-line therapy for amoebiasis and giardiasis, its use is likely to be more frequent in settings where infections associated with poor water, sanitation, and hygiene are prevalent, such as in low-income countries. Despite this, relatively limited and heterogeneous data are available regarding MTZ use in low- and middle-income countries [6–15]. Resistance to MTZ has already emerged as a concern, particularly in the treatment of diarrheal diseases caused by Giardia species and Clostridioides difficile, and may further increase due to irrational prescribing practices [16, 17]. Although MTZ is not included in the WHO watch group, its rational use warrants close attention.

While AMR can arise naturally, it is largely driven by inappropriate antibiotic use [18]. Therefore, surveillance of antibiotic consumption and the implementation of antibiotic stewardship programs, including in community settings, are essential components in addressing AMR [18]. Antibiotics are widely prescribed in primary care across low- and middle-income countries, where non-medical prescribers play a significant role in delivering healthcare, particularly in Africa [19, 20]. In this context and given the lack of studies specifically examining MTZ use in primary healthcare centers, we conducted a register-based cross-sectional study aimed at evaluating the frequency and determinants of MTZ prescribing in public and private primary healthcare centers in South Benin.

## Materials and methods

### Study type and setting

**Fig. 1:**
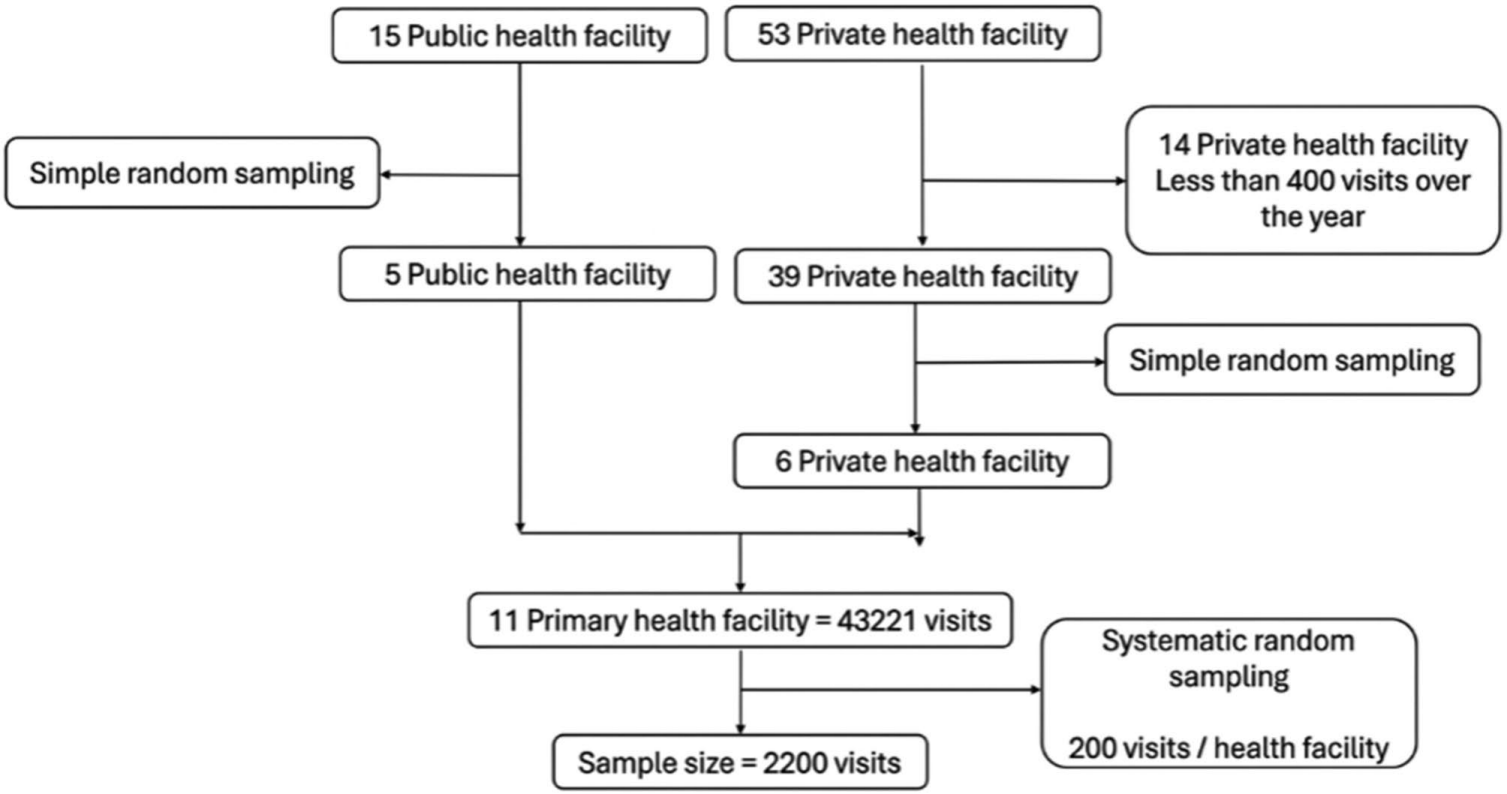
Flowchart of visit selection

This register-based cross-sectional study was conducted in public and private primary healthcare facilities in Abomey-Calavi, a city in southern Benin. All included facilities operate under the supervision of the regional public health administration (formal health sector). The study focused on facilities where prescriptions are predominantly issued by nurses. These centers have limited laboratory capacity, with malaria rapid diagnostic tests (RDTs) being the most widely available diagnostic tool. The selection of facilities is shown in the flowchart in Fig. 1.

### Sample size and inclusion criteria

The sample size was determined in accordance with the WHO guidelines for studies assessing prescribing practices in health facilities [21]. The WHO recommends collecting data from at least 600 medical visits across all facilities, with a minimum of 30 visits per selected health center. Thus, the total number of visits depends on the number of selected facilities. We elected to record 200 medical visits per healthcare facility. Eligible facilities were defined as those belonging to the formal health sector and reporting more than 400 visits per year. All public healthcare facilities met the inclusion criteria, whereas only 39 of the 53 private facilities were eligible. From these, five public healthcare centers and six private healthcare facilities were randomly selected. Based on 200 visits per facility, the final expected sample size was 2,200 medical visits.

### Sampling procedure

A multistage sampling procedure was applied. In the first stage, public and private healthcare facilities were selected as described above. In the second stage, 200 medical visits per facility were extracted from registries covering the entire year 2020. Systematic random sampling was performed using the registries of recorded visits. The sampling interval was calculated by dividing the total number of annual visits by the number of visits to be collected per facility. The total number of visits per facility was obtained from annual reports provided by the health administration. The first patient was randomly selected between the first entry in the registry and the sampling interval.

### Data collection

Data collected included demographic variables (age, sex, address), clinical information (symptoms and diagnosis), laboratory investigations (malaria RDT, blood smear, and hemogram), and therapeutic data. Six healthcare providers were trained in data collection using KoboCollect version 2.021.03. Medical visits with missing data were replaced by the next eligible visit while maintaining the predefined sampling interval. The medications analyzed were antibiotics and antimalarials, classified according to the WHO Anatomical Therapeutic Chemical classification system.

### Outcomes

The primary outcome was the prevalence of MTZ prescription.

### Independent variables

Age was recorded as a continuous variable in years, and sex as a binary categorical variable (male or female). For pediatric patients, age in months was converted to years by dividing by 12. Clinical symptoms were categorized by system and recoded as binary variables. The following categories were defined: digestive symptoms (vomiting, nausea, abdominal pain, oral thrush, or diarrhea), genitourinary symptoms (dysuria, vaginal discharge, urinary burning, urethral discharge, or metrorrhagia), skin lesions (rashes, traumatic or non-traumatic wounds, abscesses, or itching), respiratory symptoms (cough, rhinorrhea, sore throat, or dyspnea), and fever (self-reported and/or documented). Malaria was considered present if a positive RDT or blood smear was documented, or in the absence of laboratory confirmation, if the prescriber recorded malaria as the clinical diagnosis.

### Statistical analysis

Qualitative variables were summarized as counts and percentages, and quantitative variables as medians with interquartile ranges (25th–75th percentiles). Differences between the metronidazole and non-metronidazole groups were assessed using the Wilcoxon rank-sum test for quantitative variables and Fisher’s exact test for qualitative variables.

To identify determinants of metronidazole prescription, we followed a two-step modelling strategy. First, all candidate variables were simultaneously entered into a multivariable logistic regression model: age (modelled using restricted cubic splines with five knots placed at empirical quantiles), sex, healthcare sector, fever, digestive symptoms, respiratory symptoms, skin lesions, genitourinary complaints, and malaria [22]. The contribution of each predictor was assessed using likelihood ratio tests. For age, both the global effect and potential non-linearity were tested by comparing nested models. There was no missing data among the candidate predictors. Second, only variables with a statistically significant contribution were retained in the final model. The significance threshold was set at *P* < 0.05, and all tests were two-sided. Model discrimination was assessed using the C-statistic (AUC) and calibration using bootstrap-corrected calibration plots (B = 1000 resamples) [22].

As a sensitivity analysis, a mixed-effects logistic regression model with a random intercept for study site was fitted to account for potential clustering by healthcare facility. All analyses were performed in R 4.6.0.

## Results

### Characteristics of the study population and antimicrobial prescription

Among the 43,221 medical visits recorded in 2020, in total, 2,200 visits from 11 healthcare centers were analyzed, representing 5% of all visits (Fig. 1).

As shown in eTable 1 (Supplementary Material), most visits occurred in the private sector. The median age of the study population was 19 years, and 57% of patients were female. Fever was the most common symptom (67%), followed by digestive symptoms (38%) and respiratory symptoms (12%). Antimalarials were prescribed in 52% of visits. Antibacterial agents were also frequently prescribed, including penicillins (27%), MTZ (18%), ciprofloxacin (12%), and ceftriaxone (4.7%).

As shown in eTable 2 (Supplementary Material), malaria testing was frequently documented among visits with fever. A rapid diagnostic test (RDT) was documented in 1,044 of 1,453 febrile visits (71.9%), with results available for 1,008; of these, 798 (79.2%) were positive. A thick blood smear was documented in 278 of 1,453 febrile visits (19.1%), with results available for 142; of these, 98 (69%) were positive. Among visits with a malaria diagnosis, more than 80% had at least one positive test (1016 / 1214; 83.6%).

As shown in eTable 3, antimalarial monotherapy was the most common treatment pattern, accounting for 25% of visits. Overall, 20.1% of visits did not receive any antimicrobial therapy. Combination therapies were also frequent: 27.5% of visits received an antimalarial plus at least one antibiotic, while 27.4% received at least one antibiotic without antimalarial therapy.

### Characteristics of the study population and MTZ prescription

As shown in Table 1, visits with MTZ prescription were more frequently associated with digestive symptoms, skin lesions, and genitourinary symptoms compared with visits without MTZ prescription. In contrast, fever (including malaria) and respiratory symptoms were less frequent in visits with MTZ prescription. MTZ prescription was more frequent in the private healthcare sector than in the public sector.

**Table 1:** Population characteristics according to MTZ prescription in public and private healthcare facilities in South Benin, 2020

Variables	Metronidazole		*p*-value^2^
	No = 1801^1^	Yes = 399^1^	
Age	19 (6, 32)	22 (7, 33)	0.2
Sex, Female	1,041 / 1,801 (58%)	213 / 399 (53%)	0.11
Public sector	851 / 1,801 (47%)	149 / 399 (37%)	< 0.001
Fever	1,263 / 1,801 (70%)	190 / 399 (48%)	< 0.001
Digestive symptoms	545 / 1,801 (30%)	255 / 399 (64%)	< 0.001
Respiratory tract symptoms	239 / 1,801 (13%)	21 / 399 (5.3%)	< 0.001
Skin lesions	117 / 1,801 (6.5%)	50 / 399 (13%)	< 0.001
Genitourinary symptoms	14 / 1,801 (0.8%)	20 / 399 (5.0%)	< 0.001
Malaria	1,088 / 1,801 (60%)	126 / 399 (32%)	< 0.001

^1^Median (Q1, Q3); n / N (%)

The antimicrobials most frequently co-prescribed with MTZ were antimalarials, ciprofloxacin, and amoxicillin (Table 2). Antimalarial prescription was more common among visits without MTZ prescription than among those with MTZ prescription. Compared to visits without MTZ prescription, those with MTZ prescription had higher rates of co-prescription with ciprofloxacin (32% vs. 7.0%), cotrimoxazole (14% vs. 2.9%), and cloxacillin (8.0% vs. 2.4%) (Table 2).

**Table 2:** Antimicrobial prescription according to MTZ prescription in public and private healthcare facilities in South Benin, 2020

Variables	Metronidazole		*p*-value^2^
	No = 1801^1^	Yes = 399^1^	
Antimalarials	1,001 / 1,801 (56%)	153 / 399 (38%)	< 0.001
Ciprofloxacin	126 / 1,801 (7.0%)	127 / 399 (32%)	< 0.001
Aminopenicillin	489 / 1,801 (27%)	100 / 399 (25%)	0.4
Cotrimoxazole	52 / 1,801 (2.9%)	55 / 399 (14%)	< 0.001
Cloxacillin	44 / 1,801 (2.4%)	32 / 399 (8.0%)	< 0.001
Ceftriaxone	80 / 1,801 (4.4%)	23 / 399 (5.8%)	0.3
Gentamicin	90 / 1,801 (5.0%)	16 / 399 (4.0%)	0.4
Other antibiotics	76 / 1,801 (4.2%)	28 / 399 (7.0%)	0.017

^1^n / N (%)

^2^Pearson’s Chi-squared test

### Factors associated with MTZ prescription

As shown in Table 3, digestive symptoms (adjusted odds ratio [aOR], 8.86; 95% confidence interval [CI], 6.64–12.0), skin lesions (aOR, 2.33; 95% confidence interval [CI], 1.54–3.51), and genitourinary symptoms (aOR, 6.65; 95% confidence interval [CI], 3.09–14.7) were independently associated with MTZ prescription in multivariable analysis. Conversely, fever (aOR, 0.64; 95% confidence interval [CI], 0.48–0.85), malaria (aOR, 0.21; 95% confidence interval [CI], 0.15–0.28), and respiratory symptoms (aOR, 0.43; 95% confidence interval [CI], 0.26–0.70) were inversely associated with MTZ prescription. The model showed good discrimination (C-statistic = 0.81) and good calibration (see eFigure 1). Male sex was independently associated with MTZ prescription (aOR, 1.35; 95% confidence interval [CI], 1.05–1.73; *P* = 0.018). Age was excluded from the final model after likelihood ratio testing (*P* = 0.32). Healthcare sector was independently associated with MTZ prescription, with higher odds observed in the private sector than in the public sector (aOR, 2.31; 95% confidence interval [CI], 1.78–3.02).

**Table 3:** Factors independently associated with MTZ prescription in public and private healthcare facilities in South Benin, 2020

Variable	aOR (95% CI)^1^	*p*-value
Sex, Female		
Yes	—	
No	1.35 (1.05, 1.73)	0.018
Public sector		
Yes	—	
No	2.31 (1.78, 3.02)	< 0.001
Fever		
No	—	
Yes	0.64 (0.48, 0.85)	0.002
Digestive symptoms		
No	—	
Yes	8.86 (6.64, 12.0)	< 0.001
Respiratory tract symptoms		
No	—	
Yes	0.43 (0.26, 0.70)	0.001
Skin lesions		
No	—	
Yes	2.33 (1.54, 3.51)	< 0.001
Genitourinary symptoms		
No	—	
Yes	6.65 (3.09, 14.7)	< 0.001
Malaria		
No	—	
Yes	0.21 (0.15, 0.28)	< 0.001

^1^aOR: adjusted odds ratio; 95% CI: 95% confidence interval. Model adjusted for sex, health sector, fever, digestive, respiratory, skin, genitourinary symptoms, and malaria. Age was excluded after likelihood ratio test (*p* = 0.32)

### Sensitivity analyses

eTable 4 (Supplementary) presents the results of the random-effects model used to account for site-level effects. This model did not alter the conclusions of the primary analysis.

### Factors associated with MTZ prescription within sectors: subgroup analysis

To assess whether the determinants of MTZ prescription differed by sector, a subgroup analysis was conducted. As shown in eTable 5, patient characteristics were broadly similar between sectors: age distribution was comparable, females were slightly more represented in the public sector, and malaria was more frequent in the private sector. Digestive symptoms, skin lesions, and genitourinary symptoms were similarly distributed across sectors. In sector-specific multivariable analyses (eTable 6), malaria was inversely associated with MTZ prescription in both sectors, with a stronger association in the public sector than in the private sector (aOR, 0.11; 95% CI, 0.06–0.18 vs. 0.27; 95% CI, 0.18–0.39). Digestive symptoms, skin lesions, and genitourinary symptoms were positively associated with MTZ prescription in both sectors, with stronger associations observed in the public sector.

## Discussion

In this study setting, MTZ was the second most frequently prescribed antibiotic, and its use was primarily associated with digestive symptoms. In addition to clinical presentation, the private healthcare sector and male sex emerged as independent factors associated with MTZ prescription. Antimalarials and antibacterial agents were widely prescribed across visits, many of which were related to fever. Co-prescription, both between antimalarials and antibiotics and among antibiotics, was common.

Providing reliable data on antibiotic prescribing in primary care is essential. Our results indicate that MTZ prescription is common in the study area, accounting for 18% of all medical visits. In contrast, in primary care settings in France, MTZ represents less than 1.9% of antibiotic prescriptions, with similar trends observed in England [23, 24]. Thus, the proportion of MTZ prescriptions in this study was significantly higher than in high-income settings. However, country-level studies are needed for a more meaningful comparison with other countries.

Data from low- and middle-income countries remain heterogeneous. In hospital-based studies of inpatient antibiotic use, MTZ was not reported in a study conducted in South India [6], whereas it was among the most frequently prescribed antibiotics in studies from Africa [7–9] and Nepal [10]. In a tertiary hospital in Nigeria, nitroimidazoles were the second most commonly prescribed antibiotics after penicillins among outpatients [11]. In community settings, MTZ was not reported in a survey from South India [12], and ranked only seventh in Timor-Leste [13], but was commonly used to treat acute gastroenteritis in Nigeria [14], and frequently used for self-medication for respiratory symptoms in Nepal [15]. In a recent study among breastfeeding women attending primary care clinics in Uganda, MTZ was the most commonly used antibiotic, reported by nearly half of the participants [25].

This study specifically examined the determinants of MTZ prescription. Digestive symptoms, one of the most common reasons for consultation, were the primary driver of MTZ use. This high frequency of MTZ prescription likely reflects a tendency among nurses to suspect enteric infections, which are often treated with MTZ in this context. Moreover, the local treatment guidelines (ordinogrammes), in use during the study period, recommended MTZ for diarrhea persisting beyond seven days in children and for dysenteric diarrhea in adults, which may explain this practice. The frequent co-prescription of ciprofloxacin (32% of MTZ prescriptions) and cotrimoxazole (14%), both of which are deemed to treat common bacterial enteropathogens and are easily available in the study area, further supports this interpretation. This co-prescription, especially with ciprofloxacin, may increase the risk of emerging multidrug resistance among enteropathogens. However, data on the epidemiology of enteric pathogens and the effectiveness of MTZ against these pathogens in the study setting are limited. Antibiotic stewardship studies are therefore needed to determine the proportion of enteric infections caused by pathogens susceptible to MTZ, ciprofloxacin, and cotrimoxazole, and the clinical effectiveness of these agents in this context.

Our data do not enable us to clearly distinguish between urinary and genital symptoms. As the local treatment guidelines (ordinogrammes) (Additional Files), in use during the study period, recommended MTZ for genital infections (penile urethritis, vaginitis with leucorrhea, retained placenta with bloody discharge, amniotic fluid leak with fever) but not for urinary tract infections, the observed association between MTZ prescription and genitourinary symptoms is likely driven by symptoms suggestive of genital infections.

The observed association between MTZ prescription and skin lesions was unexpected. MTZ was recommended only for severe soft tissue infections and should be administered prior to referral according to local treatment guidelines (ordinogramme) (Additional Files), in use during the study period. However, most recorded skin lesions were traumatic wounds, and available data did not allow confirmation of infection. Prospective studies assessing the knowledge, attitudes, and practices of nurses regarding the management of skin infections would be valuable for optimizing antibiotic use.

In contrast, the inverse association between MTZ prescription and malaria or fever was consistent with previous findings showing that malaria diagnosis in the management of febrile illness is associated with reduced antibiotic prescribing [26]. Conversely, antibiotics like MTZ could be used as a substitute when malaria tests are negative [27].

Male sex was independently associated with MTZ prescription. This may be explained by the fact that the local treatment guidelines (ordinogrammes) (Additional Files), in use during the study period, do not recommend MTZ in breastfeeding women, which may result in lower prescription rates among women. A recent systematic review of antibiotic prescribing patterns in primary care found no consistent gender-related pattern, highlighting that gender remains an insufficiently explored factor in antibiotic prescribing [28]. Further studies are needed to better understand the role of sex in antibiotic prescribing practices.

The healthcare sector was the strongest non-clinical factor independently associated with MTZ prescription, with a larger effect size than that observed for male sex (aOR, 2.31 vs. 1.35). Overall, antibiotic prescription (eTable 2), including MTZ, was more frequent in the private sector. This finding prompted additional analyses focusing specifically on sector-related differences. Patient characteristics differed only marginally between private and public sectors with respect to MTZ prescription. In both sectors, digestive symptoms, skin lesions, and genitourinary symptoms were associated with increased MTZ use. Malaria was associated with lower odds of MTZ prescription in both sectors; however, this association was weaker in the private sector. This implies that the propensity to prescribe MTZ remains relatively high in private facilities, even during visits associated with malaria. Qualitative studies are needed to explore prescriber motivations.

The higher rate of antibiotic prescribing in the private sector may be explained by several factors. First, nurses in the public sector are typically supervised by medical doctors, whereas those in the private sector may practice with less oversight. In addition, drug dispensing in the public sector is generally regulated by a medical officer, which is not always the case in private facilities. Patients seeking care in the private sector may also be perceived as having greater financial resources, potentially encouraging increased drug prescribing. Finally, financial incentives associated with prescribing practices may differ between sectors [29, 30]. Further research is needed to determine whether the higher rate of anti-infective prescribing in the private sector represents a broader trend, particularly in West Africa. If confirmed, studies aimed at identifying the determinants of antibiotic overprescription in private healthcare settings would be of considerable importance.

This study, based on a rigorous sampling procedure, was specifically designed to provide a reliable description of antimicrobial prescribing practices by nurses in primary care outpatient settings, including both private and public facilities. The retrospective design minimized potential interference between the study objectives and prescribing behavior.

However, several limitations should be acknowledged. First, due to the retrospective design, clinical data were largely limited to basic records of patients’ complaints rather than standardized diagnoses, which were generally unavailable or unreliable except for malaria. Second, antibiotic prescription does not necessarily reflect actual antibiotic consumption, as data on drug dispensing and patient adherence were not available. Third, data collection occurred during the early phase of the coronavirus disease 2019 (COVID-19) pandemic, which may have influenced consultation rates and prescribing practices. However, chloroquine, which was incorporated into COVID-19 management in 2020, was restricted to dedicated diagnostic centers and prescribed only for confirmed cases, limiting its impact on the present findings. Finally, we did not track the number of substitutions, which may have led to selection bias. However, the registries were well completed, and visits with missing demographics or clinical presentations were infrequent.

## Conclusions

This study demonstrated that MTZ is commonly prescribed in primary care in the study area, with its use primarily driven by the presence of digestive symptoms. Further research is warranted to assess the appropriateness of MTZ indications among patients presenting with digestive complaints in this setting. An additional and unexpected finding was the higher rate of prescribing in the private healthcare sector, highlighting the need for targeted investigations to elucidate its underlying determinants.

## Supplementary Information

Below is the link to the electronic supplementary material.

Supplementary Material 1

## Data Availability

The datasets used and analyzed during the current study are available from the corresponding author on reasonable request.
