# Supplementary material for: Primary care metronidazole prescription in public and private facilities of South Benin: a register-based cross-sectional study
Source: BMC Infect Dis. 2026 Jun 9;26:1426. doi: 10.1186/s12879-026-13717-5 (PMC13435765; doi:10.1186/s12879-026-13717-5)
Supplement: Supplementary file 1 [file 12879_2026_13717_MOESM1_ESM.docx]

**Appendix**

eTable 1: Description of the study population and antimicrobial prescription in public and private healthcare facilities in South Benin, 2021

| **Variables** | **N = 2,200**^1^ |  |
| --- | --- | --- |
| Age | 19 (6, 32) |  |
| Sex, Female | 1,254 / 2,200 (57%) |  |
| Public sector | 1,000 / 2,200 (45%) |  |
| Fever | 1,453 / 2,200 (66%) |  |
| Digestive symptoms | 800 / 2,200 (36%) |  |
| Respiratory tract symptoms | 260 / 2,200 (12%) |  |
| Skin lesions | 167 / 2,200 (7.6%) |  |
| Genitourinary symptoms | 34 / 2,200 (1.5%) |  |
| Malaria | 1,214 / 2,200 (55%) |  |
| Antimalarials | 1,154 / 2,200 (52%) |  |
| Aminopenicillin | 589 / 2,200 (27%) |  |
| Metronidazole | 399 / 2,200 (18%) |  |
| Ciprofloxacin | 253 / 2,200 (12%) |  |
| Cotrimoxazole | 107 / 2,200 (4.9%) |  |
| Gentamycine | 106 / 2,200 (4.8%) |  |
| Ceftriaxone | 103 / 2,200 (4.7%) |  |
| Cloxacillin | 76 / 2,200 (3.5%) |  |
| Other antibiotics | 104 / 2,200 (4.7%) |  |
| ^1^Median (Q1, Q3); n / N (%) | | |

eTable 2: Fever, malaria, and malaria diagnostic testing in public and private healthcare facilities in South Benin, 2021

|  | **Fever** | |  | |  |
| --- | --- | --- | --- | --- | --- |
| **Variable** | **No**^1^ | **Yes**^1^ | **Overall**^1^ | **p-value**^2^ |  |
| Malaria | 181 / 747 (24.2%) | 1,033 / 1,453 (71.1%) | 1,214 / 2,200 (55.2%) | <0.001 |  |
| Rapid diagnostic test (RDT) documented | 218 / 747 (29.2%) | 1,044 / 1,453 (71.9%) | 1,262 / 2,200 (57.4%) | <0.001 |  |
| Rapid diagnostic test (RDT) positive^3^ | 105 / 190 (55.3%) | 798 / 1,008 (79.2%) | 903 / 1,198 (75.4%) | <0.001 |  |
| Thick blood smear documented | 102 / 747 (13.7%) | 278 / 1,453 (19.1%) | 380 / 2,200 (17.3%) | 0.001 |  |
| Thick blood smear positive^3^ | 27 / 39 (69.2%) | 98 / 142 (69 %) | 125 / 181 (69.1%) | >0.9 |  |
| Any test positive (RDT or Smear) | 129 / 223 (57.8%) | 887 / 1,137 (78 %) | 1,016 / 1,360 (74.7%) | <0.001 |  |
| ^1^n / N (%) | | | | | |
| ^2^Pearson's Chi-squared test | | | | | |
| ^3^Percentages for malaria diagnostic tests were calculated among patients with an available result for the respective test. Patients without an available result were excluded from the denominator. | | | | | |

eTable 3: Patterns of antimicrobial combination therapy in public and private healthcare facilities in South Benin, 2021

| **Variables** | **N = 2,200**^1^ |
| --- | --- |
| Pattern of antimicrobial combination |  |
| No antimicrobial therapy | 443 (20.1%) |
| Antimalarial only | 549 (25.0%) |
| Antimalarial + 1 antibiotic | 389 (17.7%) |
| Antimalarial + >=2 antibiotics | 216 (9.8%) |
| 1 antibiotic without antimalarial | 364 (16.5%) |
| >=2 antibiotics without antimalarial | 239 (10.9%) |
| ^1^n (%) | |

eTable 4: Factors independently associated with MTZ prescription in a mixed-effects logistic regression model accounting for center-level clustering

| **Variables** | **aOR (95% CI)** | **p-value** |
| --- | --- | --- |
| Public sector |  |  |
| Yes | — |  |
| No | 2.39 (1.48, 3.86) | <0.001 |
| Fever |  |  |
| No | — |  |
| Yes | 0.70 (0.53, 0.94) | 0.018 |
| Digestive symptoms |  |  |
| No | — |  |
| Yes | 9.10 (6.75, 12.3) | <0.001 |
| Respiratory tract symptoms |  |  |
| No | — |  |
| Yes | 0.43 (0.26, 0.71) | 0.001 |
| Skin lesions |  |  |
| No | — |  |
| Yes | 2.36 (1.55, 3.59) | <0.001 |
| Genitourinary symptoms |  |  |
| No | — |  |
| Yes | 6.89 (3.12, 15.2) | <0.001 |
| Malaria |  |  |
| No | — |  |
| Yes | 0.19 (0.14, 0.26) | <0.001 |
| Abbreviations: CI = Confidence Interval, OR = Odds Ratio | | |

eTable 5: Description of the study population according to healthcare sector in public and private healthcare facilities in South Benin, 2021

|  | **Health Sector** | | |  | |  |
| --- | --- | --- | --- | --- | --- | --- |
| **Variables** | **Public = 1000**^1^ | **Private = 1200**^1^ | **p-value**^2^ | |  |  |
| Age | 18 (6, 30) | 21 (6, 33) | 0.3 | |  |  |
| Sex, Female | 593 / 1,000 (59%) | 661 / 1,200 (55%) | 0.047 | |  |  |
| Fever | 687 / 1,000 (69%) | 766 / 1,200 (64%) | 0.016 | |  |  |
| Digestive symptoms | 371 / 1,000 (37%) | 429 / 1,200 (36%) | 0.5 | |  |  |
| Respiratory tract symptoms | 106 / 1,000 (11%) | 154 / 1,200 (13%) | 0.11 | |  |  |
| Skin lesions | 71 / 1,000 (7.1%) | 96 / 1,200 (8.0%) | 0.4 | |  |  |
| Genitourinary symptoms | 20 / 1,000 (2.0%) | 14 / 1,200 (1.2%) | 0.11 | |  |  |
| Malaria | 455 / 1,000 (46%) | 759 / 1,200 (63%) | <0.001 | |  |  |
| Any test positive (RDT or Smear)^3^ | 444 / 734 (60%) | 572 / 626 (91%) | <0.001 | |  |  |
| Antimalarials | 401 / 1,000 (40%) | 753 / 1,200 (63%) | <0.001 | |  |  |
| Aminopenicillin | 110 / 1,000 (11%) | 479 / 1,200 (40%) | <0.001 | |  |  |
| Metronidazole | 149 / 1,000 (15%) | 250 / 1,200 (21%) | <0.001 | |  |  |
| Ciprofloxacin | 59 / 1,000 (5.9%) | 194 / 1,200 (16%) | <0.001 | |  |  |
| Cotrimoxazole | 45 / 1,000 (4.5%) | 62 / 1,200 (5.2%) | 0.5 | |  |  |
| Gentamycine | 0 / 1,000 (0%) | 106 / 1,200 (8.8%) | <0.001 | |  |  |
| Ceftriaxone | 0 / 1,000 (0%) | 103 / 1,200 (8.6%) | <0.001 | |  |  |
| Cloxacillin | 42 / 1,000 (4.2%) | 34 / 1,200 (2.8%) | 0.081 | |  |  |
| Other antibiotics | 53 / 1,000 (5.3%) | 51 / 1,200 (4.3%) | 0.2 | |  |  |
| ^1^Median (Q1, Q3); n / N (%) | | | | | | |
| ^2^Wilcoxon rank sum test; Pearson's Chi-squared test | | | | | | |
| ^3^Percentages for malaria diagnostic tests were calculated among patients with an available result for the respective test. Patients without an available result were excluded from the denominator. | | | | | | |

eTable 6: Factors associated with MTZ prescription within healthcare sectors in public and private healthcare facilities in South Benin, 2021: subgroup analysis

|  | **Public sector** | **Private sector** |
| --- | --- | --- |

| **Variables** | **aOR (95% CI)** | **p-value** | **aOR (95% CI)** | **p-value** |
| --- | --- | --- | --- | --- |
| Fever |  |  |  |  |
| No | — |  | — |  |
| Yes | 0.54 (0.34, 0.84) | 0.007 | 0.75 (0.52, 1.08) | 0.13 |
| Digestive symptoms |  |  |  |  |
| No | — |  | — |  |
| Yes | 9.00 (5.63, 14.8) | <0.001 | 8.25 (5.78, 12.0) | <0.001 |
| Respiratory tract symptoms |  |  |  |  |
| No | — |  | — |  |
| Yes | 0.42 (0.15, 0.98) | 0.062 | 0.43 (0.23, 0.76) | 0.005 |
| Skin lesions |  |  |  |  |
| No | — |  | — |  |
| Yes | 3.04 (1.55, 5.87) | 0.001 | 2.08 (1.22, 3.50) | 0.006 |
| Genitourinary symptoms |  |  |  |  |
| No | — |  | — |  |
| Yes | 9.36 (3.33, 28.5) | <0.001 | 3.75 (1.15, 12.1) | 0.025 |
| Malaria |  |  |  |  |
| No | — |  | — |  |
| Yes | 0.11 (0.06, 0.18) | <0.001 | 0.27 (0.18, 0.39) | <0.001 |
| Abbreviations: CI = Confidence Interval, OR = Odds Ratio | | | | |

eFigure 1: Bootstrap-corrected calibration curve of the final model (B = 1000 resamples). The dashed diagonal represents perfect calibration. The light blue line shows apparent calibration; the dark blue line shows the bootstrap-corrected estimate.
